# Changes in Mechanical Properties of Vesicles by Mucin in Aqueous Solution

**DOI:** 10.3390/nano12203683

**Published:** 2022-10-20

**Authors:** Gaeul Lee, Kunn Hadinoto, Jin-Won Park

**Affiliations:** 1 Department of Chemical and Biomolecular Engineering, College of Energy and Biotechnology, Seoul National University of Science and Technology, Seoul 01811, Korea; 2 School of Chemical and Biomedical Engineering, Nanyang Technological University, Singapore 637459, Singapore

**Keywords:** mucin, mechanical properties, vesicles

## Abstract

The mechanical properties of vesicles were investigated as they were prepared, according to the ratio of mucin to dipalmitoylphosphatidylcholine (DPPC), using an atomic force microscope (AFM). After the confirmation of the vesicle adsorption on a mica surface, an AFM-tip deflection, caused by the interaction between the tip and the vesicle, was measured. The deflection showed that the tip broke through into the vesicle twice. Each break meant a tip-penetration into the upper and lower portion of the vesicle. Only the first penetration allowed the Hertzian model available to estimate the vesicle mechanical moduli. Two moduli reduced as the ratio of mucin to DPPC increased to 0.5, but the moduli were little changed above the 0.5 ratio. These results seem to be a platform for the effect of the mucin on the plasma-membrane anchoring and cellular signaling.

## 1. Introduction

Mucin is a group of high molecular weight, heavily glycosylated proteins produced by epithelial tissues in most animals [1]. This group has a unique structure consisting of a linear peptide backbone with densely packed oligosaccharide side chains and makes up the mucus layer on luminal surfaces of epithelial organs [2,3,4,5]. In addition, the membrane-bound-mucin includes a hydrophobic transmembrane stretch of amino acids to anchor its molecules to the plasma membrane and serves in functions of cell signaling [1]. Therefore, mucin has long been implicated in the pathogenesis of cancer, particularly adenocarcinomas [6]. Additionally, tumor-associated antigens have been identified on mucin [7].

The interactions of mucin with lipids have been investigated. The interaction was believed to reduce the diffusion of the lipid-micelles in the presence of mucin [8]. Although the mucin had no surface activity in its own right, it was likely to be present at the interface of the lipid layer [9]. The stability of the mucin structure was enhanced with the addition of lipid [10]. Specifically, the headgroups of phosphatidylcholine exhibited affinity with mucin more than the electrostatics [11]. Furthermore, the nanoparticles made with phosphatidylcholine were observed to facilitate the easier penetration into the layer with mucin compared to other nanocarriers [12].

**Figure 1:**
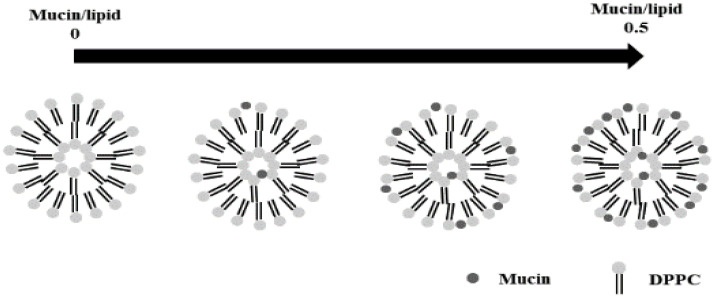
Schematic diagram of the vesicle structures with respect to the ratio of mucin/lipid.

The mechanical properties of membrane-distributed phospholipids control the vesicle-associated process and the interaction with antimicrobials [13,14]. These observations have led to the investigation of the properties [15,16]. An atomic force microscope (AFM) is capable of providing the physical properties of a surface by quantitatively measuring the interaction forces between the AFM probe and the surface [17,18,19]. Depending on the direction that the AFM probe tip moves with respect to the sample, different information is provided. The force on the approach of the tip has been available to find electrostatic and steric properties of the sample surface, and that of the retreat indicates the adhesive properties of the surface. So far, many experimental force data have been matched to the theories derived for two solid-surfaces/fluid-solid interfaces [20,21,22,23,24]. This study aimed to investigate the effect of mucin on the mechanical properties of vesicles in relation to its ratio to saturated lipid, because little is known of its metabolic mechanism in cell membranes [25]. The schematic diagram of the vesicles with respect to the ratio is shown in Figure 1. This research may provide fundamental knowledge of biological functions related to cellular processes, such as neuron response.

## 2. Materials and Methods

As biomimetic membranes, vesicles, consisting of a spherical lipid bilayer, were considered with different amounts of mucin (300 kDa, Product# M1778, Sigma Aldrich, St. Louis, MO, USA) [26]. The multi-layers of 25 mg dipalmitoylphosphatidylcholine (DPPC) from Sigma Aldrich were obtained on the bottom of a glass vial through evaporation under a dry N_2_ stream, after it was dissolved in chloroform in the glass. The layers were immersed in a 12.5 mL aqueous solution of 50 mM KCl, 10 mM Hepes, 1 mM NaN_3_, and mucin at pH 7. The suspension was vortexed four times every 20 min, at 60 °C. The suspension was poured through 100 nm pore size filters at 55 ℃ ten times [27]. The suspension was measured using dynamic light-scattering (ELS-8000, Otsuka, Tokyo, Japan) to provide the vesicle diameter (150 ± 20 nm).

AFM experiments were carried out (Nanoscope v5.12, Veeco, Santa Barbara, CA, USA). A cantilever was mounted on a liquid cell, which then was brought to the mica which was previously transferred on the top of the AFM canner. Before the transfer, the surface of the mica was peeled. The cell inside was surrounded with an elastic ring and completely filled with the suspension. The mica surface in the inside was covered entirely with the suspension at room temperature. After the coverage, the buffer solution flowed into the cell to take the extra vesicles off of the surface. The adsorption was visualized before the measurement of the interaction between the tip and the vesicle. The highest point of each bump was selected, and the deflections were measured. Only the deflection data with two penetrations were selected. Each experiment was repeated three times, and more than one hundred force-curves were acquired for each experiment.

The selected data were matched to the Hertzian contact model, which is available for the spherical point contact with the following equation [28,29].

(1) $$\begin{array}{l} \left|z-z_{0}\right|-\left(d-d_{0}\right)=\delta =A{\left(d-d_{0}\right)}^{\frac{2}{3}}\\ =0.825{\left[\frac{k^{2}(R_{\mathrm{tip}}+R_{\mathrm{ves}}){\left(1-\nu_{\mathrm{ves}}^{2}\right)}^{2}}{E_{\mathrm{ves}}^{2}R_{\mathrm{tip}}R_{\mathrm{ves}}}\right]}^{\frac{1}{3}}{\left(d-d_{0}\right)}^{\frac{2}{3}} \end{array}$$

where |*z* − *z*_0_| is the cantilever position-change [m], (*d* − *d*_0_) is the cantilever deflection [m], *δ* is the indentation [m], *A* is a parameter [m^1/3^], *k* is the AFM probe spring constant [N/m], *R_tip_* and *R_ves_* are the radiuses [m] of the tip and vesicle, *ν_ves_* is the Poisson’s ratio of the vesicle, and *E_ves_* is the Young’s modulus [Pa] of the vesicle. In this equation, three parameters, *A*, *z_0_*, and *d_0_*, are determined. *z_0_* was identified from the displacement of the boundary between distinct regions in the deflection data. Additionally, *d_0_* was found from the deflection of the boundary. Therefore, only the value of *A* needs to be found and obtained through the least-square method. This value was used to calculate *E_ves_* using Equation (1), from which the bending modulus *k_c_* [J] was estimated, using the following equation

(2) $$k_{c}=\frac{E_{\mathrm{ves}}h^{3}}{12(1-\nu_{\mathrm{ves}}^{2})},$$

where *h* corresponded to the thickness [m] of the vesicle bilayer. The value of the thickness was 5 nm [30].

## 3. Results

### 3.1. Surface Morphology

**Figure 2:**
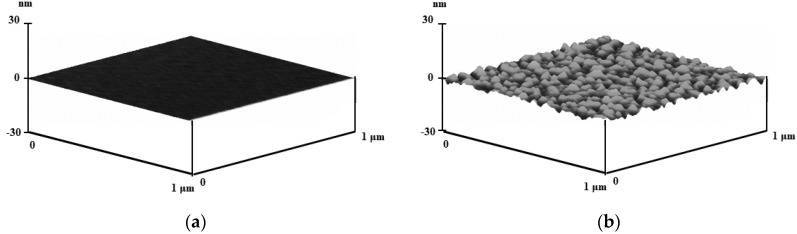
Surface morphology (**a**) before the lipid vesicle adsorption (**b**) after the adsorption (contact mode, with the tip of 15~20 nm radius and 0.02 N/m spring).

It was confirmed that the change in the ratio had little effect on the size of the vesicles. This effect was expected due to the thermodynamic property of DPPC because the structural change in the vesicle hydrophobic interior could occur only above the transition temperature of the lipids. Since two moduli could be varied upon the diameter of the vesicle, as suggested in Equation (1), the confirmation of no change in the morphology was important. The topographies are shown in Figure 1. The pre-adsorption image suggests that the roughness was less than 1 nm, even at the maximum, and a morphological bump was little found, as observed in Figure 2a. After the adsorption, the heights and the widths of most bumps were up to 10 nm and 190 ± 60 nm, respectively (Figure 2b). The roughness was calculated with the root-mean-square, as suggested in Table 1 [31]. The difference in the flatness was clearly observed between the two images.

### 3.2. Force Measurements

**Figure 3:**
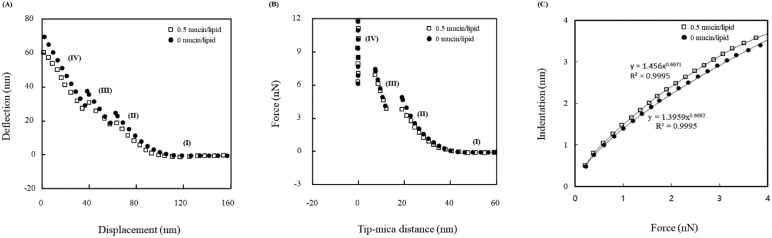
(**A**) Deflection with respect to displacement (*z* position) for vesicle at 0% mucin; (**B**) force with respect to distance based on the data in (**A**); (**C**) indentation with respect to load force based on the region (II) data of (**A**,**B**). Open square is for 0.5 mucin/lipid, and closed circle is for 0 mucin/lipid.

The behavior data included the discontinuity in the vesicle mechanical behavior. The deflection with respect to the displacement (*z* position) is presented in Figure 3A. The data in Figure 3A was used to acquire the force for the distance between the tip and the mica surface, shown in Figure 3B. In these two graphs, four regions are observed. The boundaries of each region were identified by the sudden movement of the tip. Region (I) corresponds to no tip contact with the surface of the vesicle. For region (II), the tip was in the state from its contact to the vesicle and the first penetration of the vesicle. From the data of the region (II), the Young’s modulus was estimated. Region (III) illustrates the behavior of the tip from the penetrations of the vesicle bottom bilayer. Region (IV) represented the tip behavior from the second penetration of the vesicle bilayer to the contact with the vesicle-adsorbed mica surface. The change rate of Region (IV) is theoretically about −1.0 because the AFM probe deflection is equivalent to the mica surface movement in the *z*-direction [32]. The fit of the experimental data gives a rate of −0.99. The onset of the steric region, approximately 6 nm, indicates that no vesicle was adsorbed on the tip. Therefore, the tip was placed on the mica surface after two penetrations. This analysis is supported by the comparison to the results of the experiments performed identically in pure water.

### 3.3. Theoretical Analysis

The elastic properties, shown in Figure 3B, were explained as the rate of change of force with distance [23,28]. The rate values of region (II) and Region (III) of Figure 3B are each the slope of (II) and (Ⅲ), listed with respect to the weight ratio of mucin to DPPC in Table 1. The results are average values with a less than 3% range. Each value of each region ranges from 0.65 to 0.7 and from 3.3 to 3.6 N/m, respectively. The change rates for the 0% mucin concentration were 0.7 and 3.6 N/m for each region. The fits of *δ* = *AF^b^* to the data of region (II) and Region (III) provided the estimation of b. The values of b were 0.667 (region (II)) and 0.908 (Region (III)). Considering the 3% range, the values were 0.658 to 0.676 and 0.8 to 0.935, respectively. Therefore, the values of *b* justified the elasticity of the vesicles.

## 4. Discussion

Region (II) possesses elasticity because the exponent of the deformation is 2/3 in the equation (1). The suitable fit in region (II) means that the elasticity of the vesicle within the limits of the small indentation may be addressed by the model. Region (II) in Figure 3B corresponds to the elastic deformation of the vesicle under tip compression, and thus the Hertzian model was matched to estimate the mechanical moduli. The moduli were slightly affected by the Poisson’s ratio (0 to 0.04 of the ratio increases less than 2% of moduli) [33]. Figure 3C is the indentation with respect to the load force (*F*). It indicates the consistency between the experiment results and the model. Since the force depends on the indentation in an exponential way of 0.656 to 0.676, the consistency with the Hertzian model is still secured. The calculations using Equations (1) and (2) were performed to estimate the moduli of the vesicle.

**Figure 4:**
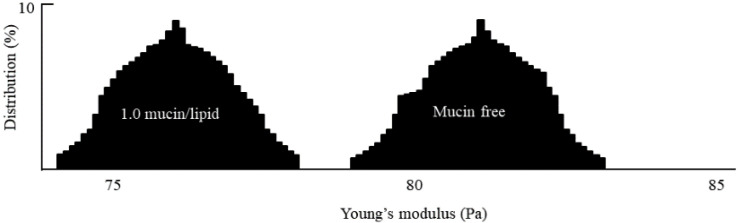
Distributions of both moduli for 1.0 mucin/lipid (**left**) and 0 mucin/lipid (**right**).

Two vesicle-moduli are summarized in Table 2 with respect to the ratio of the mucin to the DPPC. The statistical distribution was suggested in Figure 4. When compared to DPPC vesicles at 0% mucin, the moduli of the mucin-incorporated vesicles were clearly decreased. The Young’s modulus and the bending modulus of the unincorporated DPPC vesicle were 81 × 10^6^ Pa and 11.3 × 10^−19^ J, and consistent with the previous research [25,34]. Therefore, both mechanical moduli appear to depend on the mucin. The incorporation of the mucin appears to disturb the headgroup packing geometry, because the vesicles would be fused to form a planar lipid-layer through adsorption if the tail-groups were affected into unsaturated property [35]. Therefore, even though the data were indirect, the change in the behavior may be interpreted in terms of the headgroup arrangement. The more mucin there is, the lower the moduli. Additionally, the reduction was saturated at the ratios of mucin greater than 0.5. This saturation seems to be interpreted as there no longer being an association between mucin and the head group.

In the previous study, it was reported that the higher the incorporation of mucin in the portion, the higher the fluidity of the lipid layer [36]. In the research, the obvious transition from condensed to expanded was shown in the liquid-condensed phase at 36 °C. These show the tendency that is consistent with the observations in this study because the state of the vesicle corresponded to the gel phase at room temperature. In addition, it has been also suggested that the substitution effect of mucin on the stress distribution in the membrane resulted in the change in the lipid-layer phase.

## 5. Conclusions

In this research, the saturation of the modulus change was found at the mucin ratio of 0.5. This observation may be relevant to the described alterative effect, as most lipids were associated with the mucin at the ratio. In this study, the cantilever-tip behavior was interpreted to find out the mechanical behaviors of the DPPC vesicle exposed to mucin, using the Hertzian model. The mechanics were reversely proportional to the ratio of mucin to lipid until 0.5. Since the mechanical properties of the biological membranes are related to their signal transduction through their lateral diffusion and their metabolisms through their endocytosis and exocytosis, this study may provide fundamental information on biological mechanisms associated with the cellular process. Therefore, it would be interesting to investigate the relationship between mucin and the agonist-induced cells.

## Figures and Tables

**Table 1:** Change rate of the steric forces for the dipalmitoylphosphatidylcholine vesicles with respect to the ratio of mucin to lipid.

Ratio of Mucin to Lipid	0	0.1	0.3	0.5	0.7	1.0
Root-mean-square roughness (nm)	9.3 ± 0.7	9.2 ± 0.6	9.3 ± 0.6	9.3 ± 0.7	9.2 ± 0.7	9.3 ± 0.6
Change rate of 1st steric force (N/m)	0.7 ± 0.01	0.69 ± 0.01	0.67 ± 0.01	0.65 ± 0.01	0.65 ± 0.01	0.65 ± 0.01
Change rate of 2nd steric force (N/m)	3.6 ± 0.01	3.5 ± 0.01	3.4 ± 0.01	3.3 ± 0.01	3.3 ± 0.01	3.3 ± 0.01

**Table 2:** Change in Young’s modulus (*E_ves_*) and bending modulus (*k_c_*) of the dipalmitoylphosphatidylcholine vesicles, with respect to the ratio of mucin/lipid.

	Ratio of Mucin/Lipid					
	0	0.1	0.3	0.5	0.7	1.0
*E_ves_* × 10^6^ (Pa)	81 ± 2	80 ± 2	78 ± 2	76 ± 2	76 ± 2	76 ± 2
*k_c_* × 10^−19^ (J)	11.3 ± 0.3	11.2 ± 0.3	10.8 ± 0.3	10.5 ± 0.3	10.5 ± 0.3	10.5 ± 0.3

## Data Availability

Not applicable.
